# Inhibiting PDE7A Enhances the Protective Effects of Neural Stem Cells on Neurodegeneration and Memory Deficits in Sevoflurane-Exposed Mice

**DOI:** 10.1523/ENEURO.0071-21.2021

**Published:** 2021-07-03

**Authors:** Yanfang Huang, Yingle Chen, Zhenming Kang, Shunyuan Li

**Affiliations:** Department of Anesthesiology, Quanzhou First Hospital Affiliated to Fujian Medical University, Quanzhou, Fujian 362000, China

**Keywords:** sevoflurane, neural stem cells, phosphodiesterase 7A, behavioral assays, cAMP/CREB signaling

## Abstract

Sevoflurane is widely used in general anesthesia, especially for children. However, prolonged exposure to sevoflurane is reported to be associated with adverse effects on the development of brain in infant monkey. Neural stem cells (NSCs), with potent proliferation, differentiation, and renewing ability, provide an encouraging tool for basic research and clinical therapies for neurodegenerative diseases. We aim to explore the functional effects of injecting NSCs with phosphodiesterase 7A (PDE7A) knock-down in infant mice exposed to sevoflurane. The effects of PDE7A in NSCs proliferation and differentiation were determined by cell counting kit-8 (CCK-8) assay and differentiation-related gene expression assay, respectively. The effects of NSCs with modified PDE7A on mice’s long-term memory and learning ability were assessed by behavioral assays. Our data demonstrated that depleting PDE7A promoted, whereas forcing PDE7A suppressed the activation of cAMP/cAMP-response element binding protein (CREB) signaling as well as cell proliferation and neuronal differentiation of NSCs. Inhibition of PDE7A in NSCs exhibited profound improved effects on long-term memory and learning ability of mice exposed to sevoflurane. Our results for the first time show that knock-down of PDE7A improves the neurogenesis of NSCs *in vitro* and *in vivo*, and is beneficial for alleviating sevoflurane-induced brain damage in infant mice.

## Significance Statement

Our results for the first time show that knock-down of PDE7A improves the neurogenesis of NSCs *in vitro* and *in vivo* and is beneficial for alleviating sevoflurane-induced brain damage in infant mice.

## Introduction

Millions of people, including newborns, infants, and children, are subjected to general anesthesia for surgical procedures worldwide. Accumulating evidence has demonstrated that anesthetic reagents administration may have detrimental effects on the development of the brain, causing potential neurodegeneration and deficits in long-term memory and learning ability (Lu et al., 2010). Sevoflurane, a highly fluorinated methyl isopropyl ether, is commonly used as an anesthetic agent for inducing and maintaining anesthesia. Compared with other intravenous or inhalation anesthetics, sevoflurane is less irritating to the airway, a lower solubility in the blood, and sweeter smell. These features make sevoflurane a favorable choice for use in children (Eger, 2004). However, numerous studies have suggested that sevoflurane may cause neuronal apoptosis, resulting in the development of learning disability and deviant behavior (Liang et al., 2010; Lu et al., 2010; Wang et al., 2019). Therefore, it is critical to clarify the detailed effects of sevoflurane on the nervous system and behavior development, and to discover a potential therapeutic treatment to prevent or mitigate the adverse effects of sevoflurane.

Neural stem cells (NSCs), abundant in the fetal or adult nervous system, can be dissected from specific brain regions. With appropriate supplements with mitogens [e.g., epidermal growth factor (EGF), nerve growth factor (NGF), and fibroblast growth factor 2 (FGF2)] in growth media, NSCs are capable of maintaining stable proliferation, and differentiation into astrocytes, oligodendrocytes, and neurons *in vitro*. These features make NSCs an ideal cell source for regenerative medicine for various brain diseases, including stroke, spinal cord injury, and neurodegenerative diseases (Kim et al., 2013). Several preclinical studies using mouse, rat, or monkey models demonstrated that transplantation of NSCs into the brain of mice with different neurodegenerative diseases exhibited encouraging evidence of improving the functions of the brain (Lunn et al., 2011; Sakthiswary and Raymond, 2012). For example, Forotti et al. (2019) recently reported that transplantation of human induced pluripotent stem cell-derived NSCs in the ventral horn of the mouse spinal cord ameliorates neuropathological characteristics and improves the neuromuscular function and lifespan. Despite these favorable outcomes of application of NSCs for neurodegenerative disease treatment, the safety and efficacy of NSCs transplantation remain a major challenge for its clinical application in human.

Genetic modification is a useful tool to improve the function and avidity of NSCs. For example, ectopic expression of oncogene c-myc actively promotes the proliferative potential of NSCs (Swartling et al., 2015). Similarly, overexpression of Shroom family member 4 (Shrm4) activates GABA pathway in NSCs, resulting in enhanced cell growth, colony formation, anti-apoptosis, and ability to differentiate into neurons (Tian et al., 2020). The phosphodiesterase 7A (PDE7A) is an enzyme responsible for the regulation of intracellular cAMP in the central nervous system (Morales-Garcia et al., 2015). It has been revealed that administration of S14, a PDE7 inhibitor, increased NSCs proliferation, and neuronal differentiation (Chen et al., 2020). In view of those findings, we aim to investigate the functional roles of PDE7A on the NSCs cell proliferation and differentiation *in vitro*, and long-term memory and learning ability of sevoflurane-treated mice *in vivo* using genetic modification strategies.

## Materials and Methods

### Animals

C57BL/6 mice of either sex were purchased from GemPharmatech, and maintained in the standard animal care facility with free access to clean food and water. All animal experiment protocols were approved by the Ethics Committee of Quanzhou First Hospital Affiliated to Fujian Medical University.

### NSCs isolation, culture, and transplantation

The pregnant mice were euthanized, and the embryos isolated from uteruses were immersed in D-Hanks’ solution. The embryos’ brain tissues were carefully separated, followed by the removal of blood vessels and meninges. The hippocampus was carefully dissected, washed, cut into small pieces (0.2 × 0.2 mm), and digested in DMEM medium with 0.1% trypsin-EDTA, 0.15% DNAse, and 0.01% hyaluronidase in 37° for 15 min. The NSCs were washed, responded, and seeded into 12-well plate with a density of 4 × 10^4^ cells per well in DMEM/F12 medium supplied with 10 ng/ml EGF and 10 ng/ml FGF.

Two-week pups were exposed to 3% sevoflurane for 4 h, and were returned to the original cages for recovery. The main reason for the pups received NSCs injection two weeks posttreatment is that the two-week interval is an ideal period to allow the mice to recover and to familiarize themselves with new environment. We tried to minimize the potential unrelated factors, such as poor health condition or emotion, that may affect the therapeutic effect of NSCs. Thus, two weeks later, the pups were restricted in stereotaxis, and a small hole was drilled in the skull at coordinates AP, +1.7 mm, and LM, −1.0 mm. A total of 5 × 10^5^ NSCs in 100 μl of suspension was gently injected into the frontal cortex’s parenchyma with a Hamilton 80300 microsyringe. Each pup only received one-time NSCs injection.

### Morris water maze test and novel object recognition test

For Morris water maze test, a 0.6-m-high and 1.6 m in diameter circular tank containing 0.3 m high of water was incorporated in the study. A round platform (0.12 m in diameter) was placed in the center of one of the four-quadrant and submerged 1 cm beneath the water surface. The mice were trained for three times per day for a consecutive 7 d before the test. The mouse was placed into the water, and its navigation to the platform was recorded by a video camera located above the water tank. The maximum time for a mouse in the water tank was 80 s. If a mouse cannot find the platform with 60 s, the mouse was guided to the platform, and the time was recorded as 60 s. The mouse was sent back to their cages with a heat lamp after finished the test. The test was performed three times per day for 3 d, and the data were analyzed by ViewTrack video behavior tracking system (ViewPoint).

For novel object recognition test, a square plastic chamber (0.4 m long, 0.4 m wide, and 0.4 m high) was used. The detailed procedures were conducted with the published papers (Lueptow, 2017; Chen et al., 2020).

### Immunofluorescence staining

Immunofluorescence staining was performed as previously described (Chen et al., 2020). Briefly, NSCs were fixed with 4% paraformaldehyde and treated with 0.1% Triton X-100 for 30 min. Cells were blocked in 5% normal goat serum for 1 h, and the primary anti-mouse Nestin antibody (ab105398, Abcam) was added into the cells for overnight incubation at 4°. The cells were washed and further incubated with goat anti-rabbit immunoglobulin G (IgG) antibody (ab150077, Abcam) at 37° for 1 h. The cells were sealed using glycerol and were examined using an Olympus IX70 inverted fluorescence phase contrast microscope.

### Cell counting kit-8 (CCK-8) assay

CCK-8 assay (Yeasen Biotechnology) was performed according to manufactory’s instruction. Briefly, cells in 96-well plate were incubated with CCK-8 reagent for 4 h, and the optical density (OD) value of each sample was measured at the wavelength of 450 nm using GloMax Discover Microplate Reader (Promega).

### ELISA

The cAMP levels were tested by cAMP Assay kit (ab133051) following the manufactory’s instruction.

### Lentivirus packaging

Lentiviral plasmids (shPDE7A-1, shPDE7A-2, shNC, PLVX-PDE7A, and PLVX-NC) were purchased from Santa Cruz Biotechnology. Lentiviral plasmids were co-transfected with packaging plasmids into 293T cells. The cell supernatant containing lentiviral particles were collected 48 h after transfection.

### Real-time PCR (RT-qPCR)

TRIzol reagent (Invitrogen, Th.ermoFisher Scientific) was used to extract total RNA from samples according to manufactory’s instruction. The RNA was reverse transcribed into cDNA using the ReverTra Ace qPCR RT kit (FSQ-101, Toyobo Co). For gene expression analysis, the RT-qPCR was conducted using SYBR Green PCR master mix (Bio-Rad Laboratories) and analyzed with an ABI 7500 instrument (Life Technology). The relative expression levels of target genes were normalized to glyceraldehyde-3-phosphate dehydrogenase (GAPDH). The primer sequences are shown as following:

PDE7A forward: AGACGTGGAGCTATTTCCTATGA;

PDE7A reverse: CAATGTACGGATGGGAACCTC;

Microtubule-associated protein 2 (MAP-2) forward: GCC AGC CTC AGA ACA AAC AG;

MAP-2 reverse: AAG GTC TTG GGA GGG AAG AAC;

Suppressor of cytokine signaling 2 (SOCS2) forward: AGT TCG CAT TCA GAC TAC CTACT;

SOCS2 reverse: TGG TAC TCA ATC CGC AGG TTAG;

GAPDH forward: TTC ACC ACC ATG GAG AAG GC;

GAPDH reverse: GGC ATG GAC TGT GGT CAT GA.

### Western blotting

An equal amount of protein samples was loaded on an 8% SDS-PAGE gel and separated via electrophoresis. The separated proteins were transferred to a polyvinylidene difluoride (PVDF) immobilon-P membrane (Millipore). After incubated with 5% bovine serum albumin, the membranes were probed with primary antibodies in cold-room overnight and followed by incubated with second antibodies. The target protein bands were visualized using an iBind Western system (ThermoFisher Scientific). The antibodies against pCREB (Ser133, #9198), cAMP-response element binding protein (CREB; #9197), and β-actin (#4970) were from Cell Signaling Technology.

### Statistical analysis

Data values were presented as mean ± SD and were analyzed using GraphPad Prism 5. The Student’s *t* test, two-way ANOVA, or one-way followed by a *post hoc* test was used to compare the statistical difference. A value of *p* < 0.05 was considered statistically significant.

## Results

### Depletion of PDE7A promotes the cell proliferation of NSCs

**Figure 1.**
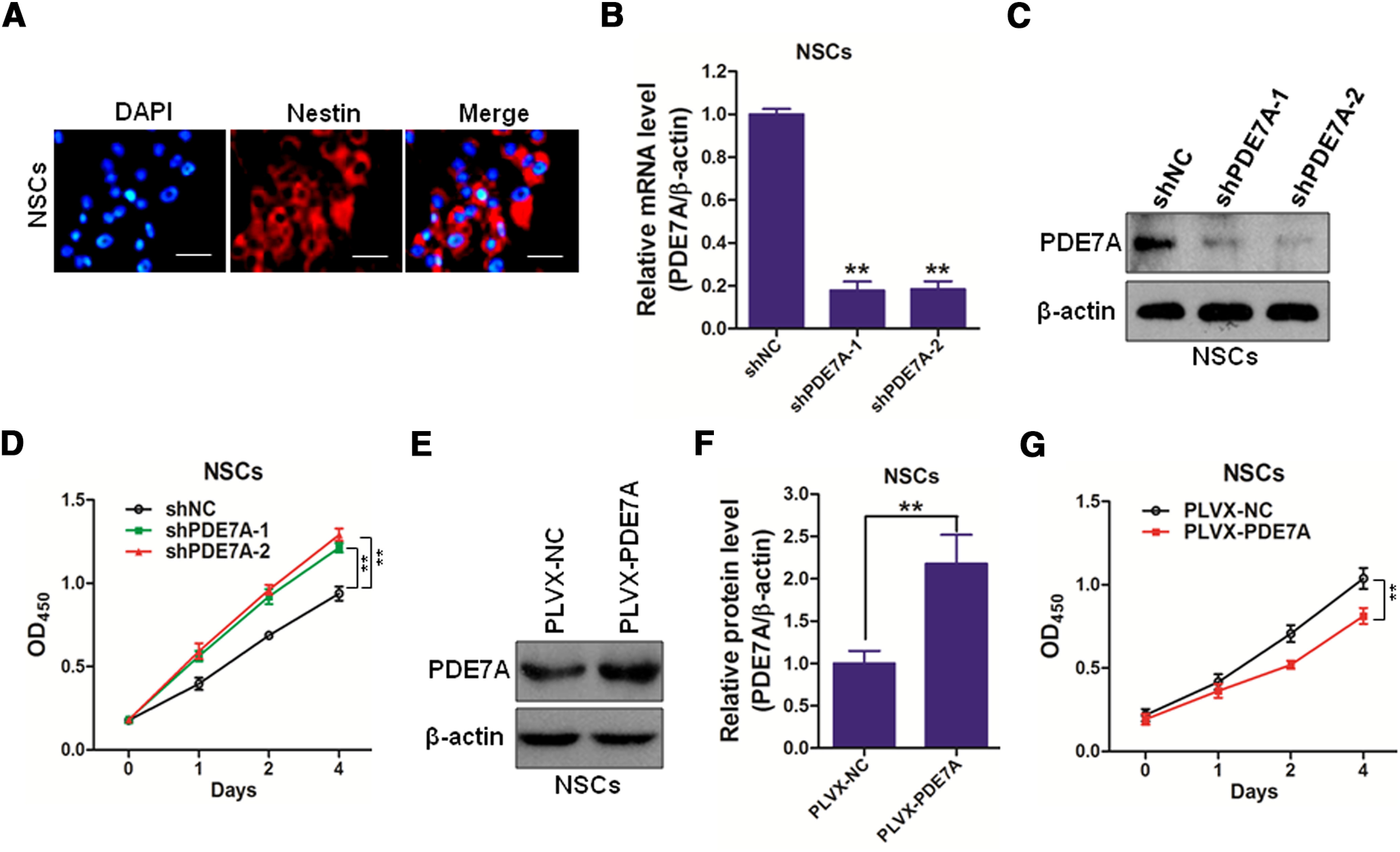
Inhibiting PDE-7A promotes cell growth of NSCs. ***A***, Nestin-positive cells of NSCs (red). DAPI, nucleus of NSCs (blue). Scale bar, 10 μm. ***B***, ***C***, The silenced efficacy of shPDE7As in mouse NSCs were determined by qRT-PCR (***B***) and Western blotting (***C***). ***D***, NSCs infected with shNC, shPDE7A-1, or shPDE7A-2 were cultured for 0, 1, 2, or 4 d, followed by CCK-8 assay. ***E***, ***F***, The overexpressing efficacy of PLVX-PDE7A in mouse NSCs were determined by Western blotting (***E***), and the OD of PDE7A was analyzed (***F***). ***G***, NSCs infected with PLVX-PDE7A were cultured for 0, 1, 2, or 4 d, followed by CCK-8 assay; **p <* *0.01.

To confirm the purity of isolated NSCs, the expression of nestin, an NSCs marker, was assessed by immunofluorescent staining. The results showed that almost all cells were positive for nestin, suggesting the successful establishment of NSCs *in vitro* (Fig. 1*A*). To investigate the functional role of PDE7A on NSCs proliferation, NSCs were infected with two lentivirally shRNA (shPDE7A-1, and shPDE7A-2) specifically against PDE7A, but not PDE7B. As showed in Figure 1*B*,*C*, transfection of shPDE7A-1 or shPDE7A-2 resulted in a profound decrease in the mRNA and protein levels of PDE7A in NSCs, as evidenced by RT-qPCR and Western blotting, respectively (Fig. 1*B*,*C*). We observed that PDE7A depletion enhanced NSCs cell proliferation (Fig. 1*D*). On the contrary, NSCs were transduced with lentivirus carrying PDE7A or non-sense control (NC), and overexpression of PDE7A in NSCs was confirmed by Western blot analysis (Fig. 1*E*,*F*). We found that forced PDE7A attenuated the cell proliferation of NSCs (Fig. 1*G*). These data suggested that PDE7A plays a negative role in the cell growth of NSCs.

### Suppression of PDE7A enhances the cell differentiation of NSCs

**Figure 2.**
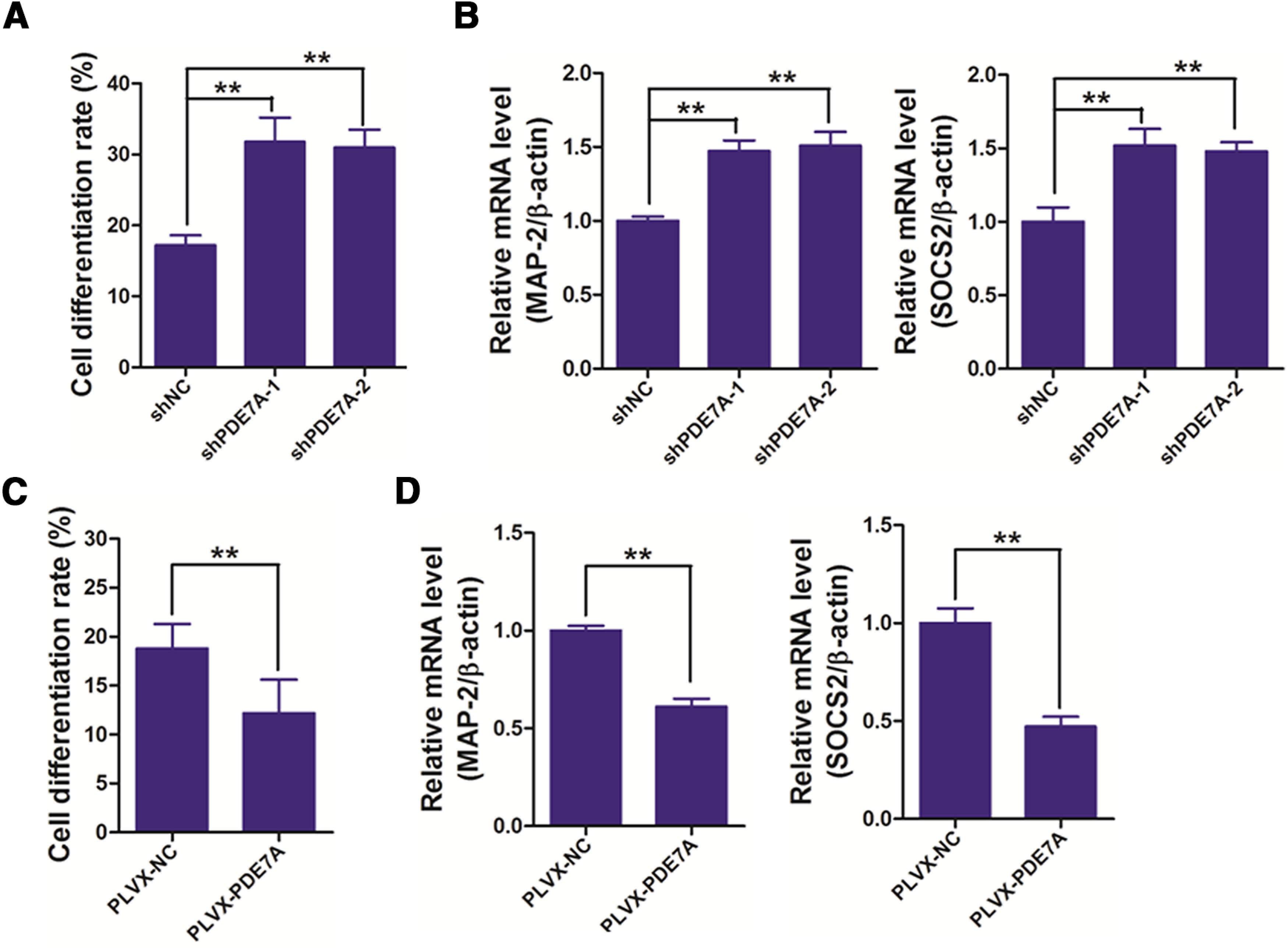
Inhibiting PDE-7A promotes cell differentiation of NSCs. ***A***, NSCs infected with shNC, shPDE7A-1, or shPDE7A-2 were cultured for two weeks, and then cell-differentiation rate was measured. ***B***, NSCs infected with shNC, shPDE7A-1, or shPDE7A-2 were cultured for two weeks, and then cells were prepared for measuring the mRNA expression of cell differentiation-related genes, including MAP-2 and SOCS2. ***C***, NSCs infected with PLVX-PDE7A were cultured for two weeks, and then cell-differentiation rate was measured. ***D***, NSCs infected with PLVX-PDE7A were cultured for two weeks, and then cells were prepared for measuring the mRNA expression of cell differentiation-related genes, including MAP-2 and SOCS2; **p <* *0.01. Extended Data Figure 2-1 is supporting Figure 2.

To study the effect of PDE7A on NSCs differentiation, we applied the same strategies in Figure 1 to knock-down of or overexpression of PDE7A in NSCs. The differentiated NSCs generally present prolonged fusiform, and continuously growing spores. We observed that silencing PDE7A promoted, whereas enhancing PDE7A inhibited cell differentiation of NSCs (Fig. 2*A*,*C*). The results were further confirmed by detection of MAP-2 and SOCS2, two NSCs differentiation markers, in the NSCs. As illustrated in Figure 2*B*,*D*, knock-down of PDE7A induced, whereas overexpression of PDE7A decreased the expression of MAP-2 and SOCS2 in NSCs. The findings were further confirmed by immunostaining against MAP-2 (Extended Data Fig. 2-1*A*,*B*). These data implied that PDE7A overexpression blocks NSCs differentiation.

10.1523/ENEURO.0071-21.2021.f2-1 Extended Data Figure 2-1 Immunostaining of the differentiated cells with MAP2 (***A***, ***B***). The mouse monoclonal anti-MAP2 antibody and Alexa Fluor 488 goat anti-mouse secondary antibody were used for detection. Counterstaining was done with Hoechst, and images were acquired using a Leica ILMD LED inverted fluorescence microscope. Download Figure 2-1, TIF file.

### Silencing PDE7A actives cAMP/CREB signaling

**Figure 3.**
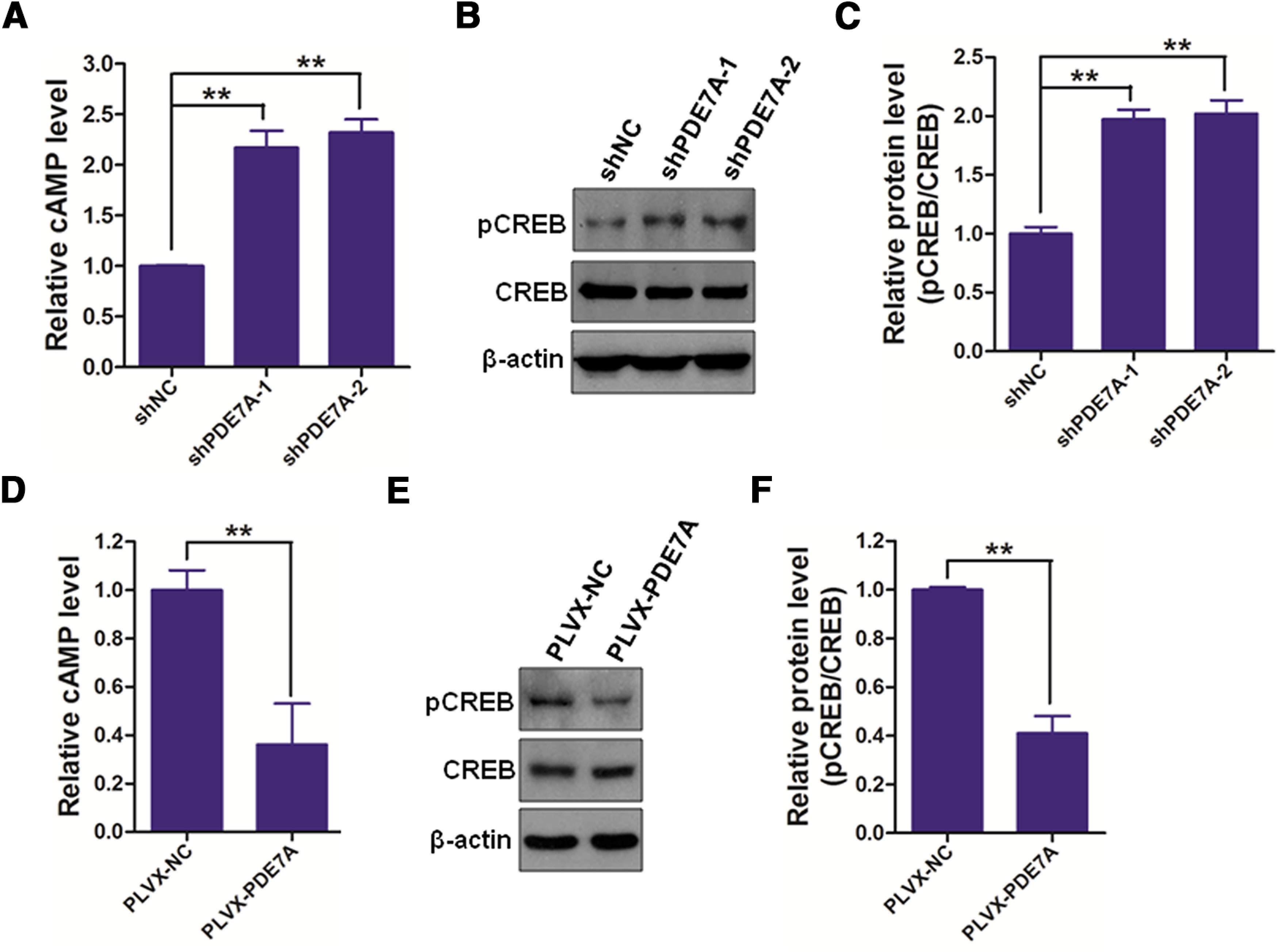
Inhibiting PDE-7A activates cAMP/CREB signaling in NSCs. ***A***, The cAMP levels in NSCs infected with shNC, shPDE7A-1, or shPDE7A-2 were determined using a mouse cAMP ELISA kit. ***B***, ***C***, NSCs infected with shNC, shPDE7A-1, or shPDE7A-2 were prepared for Western blotting (***B***), and the OD of pCREB was analyzed (***C***). ***D***, The cAMP levels in NSCs infected with PLVX-NC or PLVX-PDE7A were determined using a mouse cAMP ELISA kit. ***E***, ***F***, NSCs infected with PLVX-NC or PLVX-PDE7A were prepared for Western blotting (***E***), and the OD of pCREB was analyzed (***F***); **p <* *0.01.

cAMP/CREB signaling is known to play an essential role in learning and long-term memory (Kandel, 2012). To explore the effect of PDE7A on the activation of cAMP/CREB signaling, NSCs were again subjected to shPDE7A-1/2 or shNC, and PLVX-PDE7A or PLVX-NC transduction. The results demonstrated that PDE7A suppression substantially increased, while PDE7A augmentation significantly decreased cAMP (Fig. 3*A*,*D*) and pCREB (Fig. 3*B*,*C*,*E*,*F*) expression in NSCs, suggesting inhibition of PDE7A promotes cAMP/CREB signaling activation.

### Transplantation of NSCs/shPDE7A enhances the long-term memory of sevoflurane-exposed mice

**Figure 4.**
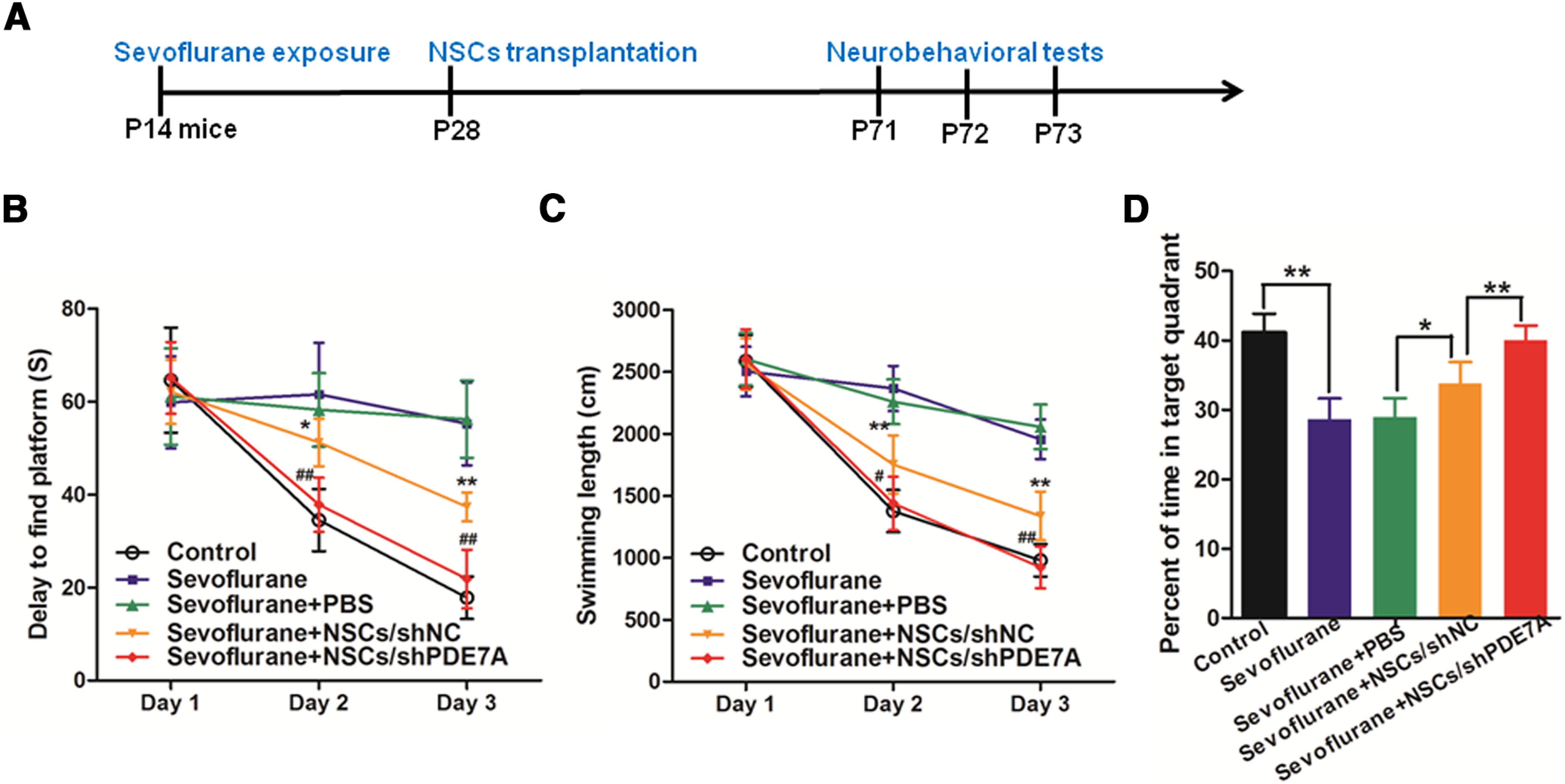
NSCs attenuates sevoflurane-induced learning and memory defects, and silenced PDE7A enhances the effects of NSCs. ***A***, Experimental design. ***B***, ***C***, Eight weeks after sevoflurane exposure, spatial learning and memory were examined by Morris water maze test on day 1 (P71), day 2 (P72), and day 3 (P73). ***B***, Delay time to reach the platform of indicated groups; *p <* *0.05, **p <* *0.01 compared with sevoflurane group; ##*p *<* *0.01 compared with sevoflurane+NSCs/shNC. ***C***, Swimming path length before reaching the platform of indicated groups; **p <* *0.01 compared with sevoflurane group; #*p *<* *0.05, ##*p *<* *0.01 compared with sevoflurane+NSCs/shNC. ***D***, Percent of time spent in the target quadrant in a probe test of indicated groups; *p <* *0.05, **p <* *0.01; *n* = 8 for each time point.

To determine whether PDE7A inhibition promotes the cellar function of NSCs *in vivo*, the two-week-aged mice were exposed to sevoflurane, and then received PBS, NSCs/shNC or NSCs/shPDE7A transplantation 14 d after sevoflurane exposure (Fig. 4*A*). The long-term memory ability of mice was assessed by the Morris water maze test. As revealed in Figure 4*B*,*C*, after the first day of training, mice from all groups exhibited comparable time (60 s) to find the platform and length of swimming (2500 cm). On days 2 and 3 after training, the normal control mice spent 38 s (day 2) and 20 s (day 3), as well as swan 1250 cm (day 2), and 1000 cm (day 3) to find the platform. However, sevoflurane-exposed and sevoflurane+PBS mice spent the amount of time (58 s on days 2 and 3) and slightly decreased swimming length (2250 cm on day 2, and 2000 cm on day 3), suggesting sevoflurane exposed significantly impaired long-term memory of mice (Fig. 4*B*,*C*). The NSCs/shNC transplanted sevoflurane-exposed mice spent less time and swam less length to find the platform compared with sevoflurane-exposed mice, suggesting NSCs/shNC transplantation partially improved the neural function of sevoflurane-exposed mice (Fig. 4*B*,*C*). Strikingly, the NSCs/shPDE7A transplanted sevoflurane-exposed mice spent similar time and swam comparable length to find the platform compared with normal control mice (Fig. 4*B*,*C*).

Furthermore, mice subjected to sevoflurane exposure spent ∼30% less time in the targeted quadrant compared with normal control mice. Sevoflurane-exposed mice with NSCs/shNC transplantation, but not PBS injection, stayed longer (20% more time) in the targeted quadrant than in control mice. Furthermore, NSCs/shPDE7A transplantation further improved the long-term memory healing effect of NSCs/shNC on sevoflurane-exposed mice, as evidenced by comparable time in the targeted quadrant between sevoflurane+NSCs/shPDE7A mice, and normal control mice (Fig. 4*D*).

### Transplantation of NSCs/shPDE7A promotes the learning ability of sevoflurane-exposed mice

**Figure 5.**
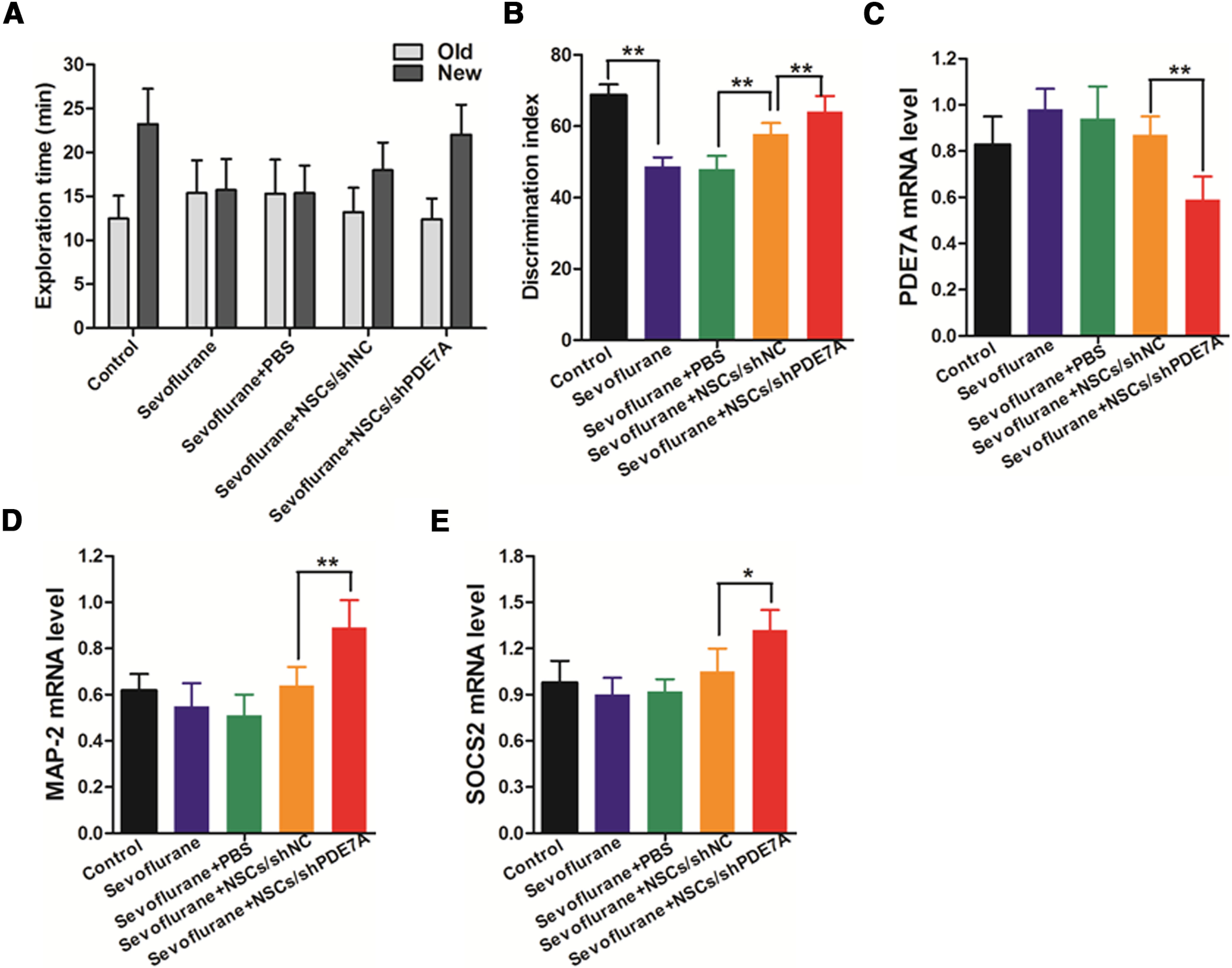
NSCs attenuates sevoflurane-induced deterioration of recognition memory, and silenced PDE7A enhances the effects of NSCs. ***A***, Exploration time of indicated group spent with an old object and new object. ***B***, Discrimination index of recognizing the new versus old object of an indicated group; *n* = 8 for each group; **p <* *0.01. ***C–E***, The mRNA levels of PDE7A, MAP-2 and SOCS2 in brain tissues were measured by RT-qPCR. β-Actin was used as an internal control; *p <* *0.05, **p <* *0.01. Extended Data Figure 5-1 is supporting Figure 5.

To further assess the learning and memory in those mice, mice were subjected to the novel object recognition test. As displayed in Figure 5, the normal healthy mice spent 80% longer time on the new object than on the old object. Both sevoflurane and sevoflurane+PBS mice spent equivalent amounts of time on new and old objects, with the same discrimination index value, which was considerably low compared with normal control (Fig. 5*A*,*B*). Both NSCs/shNC and NSCs/shPDE7A transplantation resulted in a prolonged time on the new object than on the old one. Of note, the NSCs/shPDE7A transplantation yielded an improved memory rescue effect than NSCs/shNC transplantation, as evidenced by the discrimination index value in sevoflurane+NSCs/shPDE7A mice was higher than that in sevoflurane+NSCs/shNC mice (Fig. 5*A*,*B*). Furthermore, the mRNA levels of PDE7A, MAP-2, and SOCS2 in brain tissues were measured by RT-qPCR. As manifested in Figure 5*C–E*, the expression levels of PDE7A were significantly decreased, whereas the expression levels of MAP-2 and SOCS2 were markedly upregulated in the brain tissues of sevoflurane+NSCs/shPDE7A mice when compared with that from sevoflurane+NSCs/shNC mice. No significant difference of PDE7A, MAP-2, and SOCS2 expression was observed in the other four groups. In addition, the Western blot analysis showed that the expression of pCREB was also upregulated in the brain tissues of sevoflurane+NSCs/shPDE7A mice when compared with that from sevoflurane+NSCs/shNC mice (Extended Data Fig. 5-1).

10.1523/ENEURO.0071-21.2021.f5-1 Extended Data Figure 5-1 Western blot analysis of pCREB expression in brain tissues. The protein levels of pCREB in brain tissues were measured by Western blotting. β-Actin was used as a loading control. Download Figure 5-1, TIF file.

## Discussion

In recent years, Gil’s lab and others have published a series of papers to reveal the critical role of PDE7 in regulating various biological functions of NSCs (Miró et al., 2001; Sasaki et al., 2002; Morales-Garcia et al., 2011, 2017; Redondo et al., 2012; Perez-Gonzalez et al., 2013; Liu et al., 2015). PDE7 is a cAMP-specific PDEs, and the latter comprises a family of 21 members with distinct sequence homology, cellular distribution, and expression pattern in central nervous systems. Strikingly, several studies have exhibited that suppression of PDE7 using different PDE7 inhibitor or shRNA, exerts potent neuroprotective and anti-inflammatory effects on animal models of neurodegenerative disorders (Morales-Garcia et al., 2011, 2017; Redondo et al., 2012; Perez-Gonzalez et al., 2013; Liu et al., 2015). Administration of inhibitor of PDE7 (e.g., S14, or 3-phenyl-2,4-dithioxo-1,2,3,4-tetrahydroquinazoline) can protect neurons against adverse chemical-induced or inflammatory-induced cell death, promote NSCs generation, migration, and differentiation, ameliorate neuron system damage, improved learning and behavioral outcome of animal models of neurodegenerative diseases, including stroke, Alzheimer’s disease (AD), and Parkinson’s disease (PD; Morales-Garcia et al., 2011, 2017; Redondo et al., 2012; Perez-Gonzalez et al., 2013; Liu et al., 2015). In line with these findings, instead of using PDE7 inhibitor, we knock-down of endogenous PDE7A using shPDE7A-1/2 in NSCs. We found that depletion of PDE7A enhanced NSCs cell proliferation and differentiation. More importantly, we forced expression of PDE7A using lentiviral transduction in NSCs, and we revealed that overexpression profoundly inhibited NSCs cell proliferation and differentiation. Our data further confirmed the previous findings, highlighting that PDE7A plays a negative role in NSCs cell proliferation and differentiation.

cAMP levels have been shown to play a pivotal role in neuronal differentiation, neuroprotection, and neuroinflammatory response (Lee, 2015). Several studies suggested that modulation of cAMP levels could trigger the pathologic features of neurons, and delay the progression of neurodegenerative disorders (Inda et al., 2017). CREB is a nuclear transcription factor that binds to cAMP response element of the targeted genes’ promoter and regulates the expression of genes that participate in neuronal function and survival (Wang et al., 2018). The activation of cAMP/CREB signaling pathway has been demonstrated to be closely associated with various neuronal functions, including cell proliferation, apoptosis, differentiation, neurogenesis, and neural plasticity. Numerous studies reported that cAMP/CREB signaling pathway plays an essential role for maintaining normal neuronal functions, memory formation and retention, healthy mental status, and normal behavior. Disruption of cAMP/CREB signaling pathway has been reported to be associated with schizophrenia, AD, and PD (Sakamoto et al., 2011). Studies from Gil’s lab have presented that inhibition of PDE7 induced the activation of cAMP/PKA signaling pathway and subsequently activated CREB by phosphorylation at Ser133 (Morales-Garcia et al., 2011). In accordance with these observations, we confirmed that knock-down of PDE7A enhanced, whereas overexpression of PDE7A inhibited the activity of cAMP/CREB signaling in NSCs. cAMP signaling has been associated with neuroplasticity and protection, and influencing their levels in the cell by inhibition of PDEs has become a studied target for treatment in a wide array of disorders, including neurodegenerative disorders (Bollen and Prickaerts, 2012). Therefore, we believe that inhibition of PDE7A leads to activation of cAMP/PKA signaling pathway, which is beneficial for neural cell survival, proliferation, and differentiation (Neumann et al., 2002; Nikulina et al., 2004; Perez-Gonzalez et al., 2013).

NSCs are known as the “seed” cells of the central nervous system. They are a group of cells that are capable of self-proliferation, renewing, and differentiation into neurons and glia during central nervous system development (Lu et al., 2003). The limited source for NSCs hampered the application of NSCs in the clinical field in the past decades. With the rapid advances in the stem cell researches, several pluripotent stem cells, such as induced pluripotent stem cells, mesenchymal stem cells (MSCs), and embryonic stem cells (ESCs) can be induced to differentiate into NSCs, making the use of NSCs for treatment of neurodegenerative diseases becomes possible (Marsh and Blurton-Jones, 2017). Remarkably, accumulating studies have reported the encouraging results of a significant functional benefit after NSCs transplantation in various animal models (Hattiangady and Shetty, 2011; Yi et al., 2013). Furthermore, several strategies have been explored to improve the therapeutic potential of NSCs. For instance, genetically modified NSCs to produce neurotrophin-3 induce secreting other growth factors and promote host neural repair (Lu et al., 2003). Similarly, NSCs stable-expressing neurotrophies, such as glial cell-derived neurotrophic factor (GDNF), brain-derived neurotrophic factor (BDNF), and NGF, exhibited remarkably improved proliferation, survival, and functional recovery in different neurologic disease animal models (Chen et al., 2017). Morales-Garcia et al. (2011) showed that application of S14, a PDE7 inhibitor, exerts potent anti-inflammatory and neuroprotective effects, and promotes dopaminergic neuron generation in mice with PD. To extend this finding, we used shRNA to specifically knock-down PDE7A in NSCs. Importantly, we found that NSCs/shPDE7A transplantation substantially improved the long-term memory and learning ability in sevoflurane-exposed mice. Compared with inhibitor treatment, genetic modification has apparent advantages. Repetitive administration of inhibitor is labor-consuming, and may potentially cause adverse side effects to the recipients. In the next step, we aim to exploit the depletion of PDE7A using clustered regularly interspaced short palindromic repeats (CRISPR)-knock-out technology in NSCs and apply the engineered NSCs-CRISPR-PDE7A to mice exposed to sevoflurane or mice with PD or AD symptoms.

In conclusion, our results confirmed that PDE7A is a crucial target in the regulation of NSCs functions. Suppression of PDE7A in NSCs may serve as a new therapeutic strategy for the treatment of sevoflurane-induced brain impairment in newborns, infants, and children.

## Synthesis

Reviewing Editor: Susanne Wolf, Charite University Hospital Decisions are customarily a result of the Reviewing Editor and the peer reviewers coming together and discussing their recommendations until a consensus is reached. When revisions are invited, a fact-based synthesis statement explaining their decision and outlining what is needed to prepare a revision will be listed below. The following reviewer(s) agreed to reveal their identity: Xian Zhang, Devi Krishna Priya Karunakaran.

Dear Authors, both reviewers feel that you addressed their comments very well. However, one reviewer raised the question for additional data (see below). Please let me know, whether you can perform the experiment with existing materials (RNA/Protein) or if you would need to perform additional in vivo experiments. If you could add the requested data without performing additional in vivo experiments, please do so. The revised version will not go out to the reviewers again. It will be an editorial review only.

The authors present an important finding to show that PDE7A knockdown in neural stem cells (NSC) can be used to treat infants/kids with sevoflurane-related brain disorder in this revised manuscript. They show that the suppression of PDE7A in NSC transplant in mice in conjunction with sevoflurane treatment ameliorates the learning memory deficits in mice concurrent with decreased PDE7A and increased MAP2 and SOCS2 levels. Although the authors have addressed the major concerns, there is one concern that could be addressed to accept this manuscript.

Major revision:

1. Authors have included mRNA levels for PDE7A knockdown and increase in SOCS2 and MAP2 levels, but could also include mRNA and protein levels of cAMP, pCREB levels in the in vivo study as a convincing way to support the proposed mechanism of action of PDE7A knockdown.
