# Supplementary figures and images for: Inhibiting PDE7A Enhances the Protective Effects of Neural Stem Cells on Neurodegeneration and Memory Deficits in Sevoflurane-Exposed Mice
Source: eNeuro. 2021 Jul 3;8(4):ENEURO.0071-21.2021. doi: 10.1523/ENEURO.0071-21.2021 (PMC8266220; doi:10.1523/ENEURO.0071-21.2021)

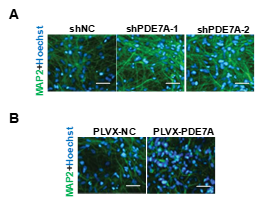

Supplement: Extended Data Figure 2-1 — Immunostaining of the differentiated cells with MAP2 (A, B). The mouse monoclonal anti-MAP2 antibody and Alexa Fluor 488 goat anti-mouse secondary antibody were used for detection. Counterstaining was done with Hoechst, and images were acquired using a Leica ILMD LED inverted fluorescence microscope. Download Figure 2-1, TIF file. [file enu-eN-NWR-0071-21-s02.tif]

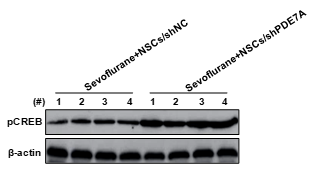

Supplement: Extended Data Figure 5-1 — Western blot analysis of pCREB expression in brain tissues. The protein levels of pCREB in brain tissues were measured by Western blotting. β-Actin was used as a loading control. Download Figure 5-1, TIF file. [file enu-eN-NWR-0071-21-s03.tif]
